# Endothelial-Fibroblast Organoids Generated in Hydrogel Microchamber Arrays: A Translational Model of Vascular Dysfunction in Cardio-Kidney-Metabolic Disease

**DOI:** 10.1159/jvr/adiag003

**Published:** 2026-09-30

**Authors:** Tali Zitman-Gal, Maria Sobolev, Anna Talash, Shelly Tartakover-Matalon, Guy Topaz, Jacob Chen, David Pereg, Gloria Rashid, Naomi Nacasch, Arik Dahan, Mordechai Deutsch, Keren Cohen-Hagai

**Affiliations:** a Department of Nephrology and Hypertension, Meir Medical Center, Kfar Saba, Israel; b Gray Faculty of Medical and Health Sciences, Tel Aviv University, Tel Aviv, Israel; c Autoimmune Research Laboratory, Meir Medical Center, Kfar Saba, Israel; d Hospital Managment, Meir Medical Center, Kfar Saba, Israel; e Department of Cardiology, Meir Medical Center, Kfar Saba, Israel; f Department of Clinical Pharmacology, School of Pharmacy, Faculty of Health Sciences, Ben-Gurion University of the Negev, Beer-Sheva, Israel

**Keywords:** Endothelial cell, Spheroid, Organoid, Hydrogel microchambers, Cardio-kidney-metabolic disorder

## Abstract

**Introduction:**

Cardio-kidney-metabolic (CKM) disease comprises interconnected cardiovascular, renal, and metabolic disorders in which endothelial dysfunction drives microvascular and macrovascular injury. Although novel treatments improve clinical outcomes, the cellular and molecular mechanisms underlying these effects remain incompletely understood. Addressing this gap requires experimental systems that recapitulate the structural and functional complexity of the vascular microenvironment. The objective of this study was to establish and characterize a three-dimensional (3D) endothelial and fibroblast co-culture platform for controlled investigation of endothelial morphogenesis, stromal interactions, and responses to metabolic and pharmacological stimuli.

**Methods:**

We developed a hydrogel microchamber (HMC) array platform enabling reproducible formation of endothelial spheroids and extracellular matrix-embedded organoids. Human umbilical vein endothelial cells were cultured alone or with NIH3T3 fibroblasts, allowing direct comparison of growth configurations within a defined 3D microenvironment. Morphometric parameters were quantified longitudinally following glucose exposure to model diabetic-like conditions and treatment with a glucagon-like peptide-1 receptor agonist/insulin combination (Xultophy).

**Results:**

The HMC platform supported reproducible formation of monolayers, scaffold-free spheroids, and matrix-embedded organoids. Fibroblast co-culture enhanced endothelial organization and structural complexity. Glucose exposure induced time-dependent increases in organoid size and reduced circularity, indicating structural remodeling. Xultophy treatment moderated these effects and preserved higher circularity. Sequential stimulation revealed order-dependent responses, with Xultophy partially attenuating glucose-induced expansion. Organoids exhibited stronger responses than spheroids, underscoring the role of extracellular matrix context.

**Conclusion:**

The HMC system provides a reproducible platform for studying endothelial morphogenesis and stromal regulation in physiologically relevant 3D environments, enabling controlled investigation of endothelial responses to metabolic stress and therapeutic modulation.

## Introduction

The growing recognition of shared pathophysiological mechanisms linking metabolic disorders, chronic kidney disease, and cardiovascular disease has led to the cardiovascular-kidney-metabolic (CKM) framework [1]. Within this continuum, endothelial dysfunction is a central pathogenic process. The vascular endothelium maintains homeostasis through regulation of vascular tone, angiogenesis, inflammation, and hemostasis, and its disruption is strongly associated with accelerated atherosclerosis, vascular calcification, and increased cardiovascular morbidity and mortality in chronic kidney disease and metabolic disease [1–5].

Diabetes mellitus is a major contributor to endothelial dysfunction within the CKM continuum. Chronic hyperglycemia induces metabolic, oxidative, and inflammatory changes in endothelial cells, including reduced proliferation, increased apoptosis, and activation of pro-inflammatory pathways [6–9], ultimately impairing angiogenesis and vascular homeostasis. In parallel, hyperglycemia alters stromal cell function, particularly fibroblast metabolism, extracellular matrix (ECM) production, and paracrine signaling, thereby disrupting endothelial-stromal communication and vascular repair processes [10, 11].

Glucagon-like peptide-1 receptor agonists (GLP-1RAs) have emerged as an important therapeutic class in diabetes, with demonstrated cardiovascular and renal benefits [12, 13]. Beyond glycemic control, these agents modulate vascular inflammation and endothelial signaling [14–16]. However, the mechanisms by which metabolic stress and GLP-1RA-based therapies influence endothelial organization and vascular remodeling remain incompletely understood, particularly in physiologically relevant three-dimensional (3D) microenvironments.

Technological advances in cell culture have enabled a transition from traditional two-dimensional (2D) monolayer systems to 3D culture platforms that more closely approximate native cellular environments [17, 18]. Although conventional 2D cultures remain widely used due to their simplicity and accessibility, they fail to reproduce the spatial organization, cell-cell communication, and matrix-dependent signaling present in living tissues. Increasing evidence indicates that endothelial responses to metabolic and pharmacological stimuli differ substantially between 2D and 3D environments, highlighting the importance of spatial context for endothelial function [17–19].

Among 3D models, endothelial spheroids and organoids represent complementary approaches for studying vascular organization. Endothelial spheroids are widely used to investigate angiogenic sprouting, lumen formation, and collective migration. These structures develop a characteristic architecture consisting of an outer differentiated cell layer surrounding a central core, resulting in enhanced survival and altered responsiveness to angiogenic stimuli compared with single-cell cultures [19]. In parallel, endothelial organoid systems embedded within ECM-rich matrices such as Matrigel enable collective migration, branching, and lumen-like organization, thereby recapitulating aspects of vascular morphogenesis that are limited in scaffold-free spheroid systems [20–22]. However, despite these advances, many existing 3D culture approaches suffer from limited reproducibility, heterogeneity, and scalability. Conventional spheroid-generation techniques often produce variable aggregate sizes and provide limited control over spatial organization, while matrix-based systems exhibit batch-to-batch variability and structural instability due to the poorly defined composition of ECM materials [17, 18, 23–25].

Micro-engineered culture systems offer an opportunity to overcome these limitations by enabling precise control over cellular organization and microenvironmental parameters. Microfabrication approaches, including microwell arrays, microfluidic devices, and hydrogel patterning, have significantly advanced the field of 3D cell culture [26, 27]. Among these, hydrogel microchamber (HMC) arrays combine physical confinement, optical accessibility, and compatibility with standard multiwell plate formats. This enables uniform generation of microtissues and supports high-content imaging and multiplexed drug screening at single-microtissue resolution [28, 29]. Importantly, such systems allow controlled investigation of endothelial responses under physiologically relevant conditions that incorporate spatial and biochemical complexity.

To address these challenges, we developed a HMC platform designed to support controlled formation of endothelial microtissues under defined conditions. The system enables precise regulation of microtissue size, geometry, and cellular composition while supporting both endothelial monoculture and endothelial-fibroblast co-culture configurations. By enabling parallel generation of endothelial monolayers, spheroids, and ECM-embedded organoids within a single experimental framework, this platform provides a versatile and reproducible system for investigating endothelial morphogenesis and stromal regulation in physiologically relevant 3D microenvironments.

The aim of this study was to establish and characterize this HMC platform and to demonstrate its utility for studying endothelial-stromal interactions and vascular responses under diabetic-like conditions, including glucose exposure and pharmacological modulation with GLP-1 receptor agonists. This approach enables direct comparison of multiple endothelial growth modalities within a unified system and provides a robust platform for investigating structural and functional vascular responses to metabolic and therapeutic perturbations in 3D environments.

## Methods

### Materials and Reagents

The reagents and materials are detailed in the online supplementary materials (for all online suppl. material, see https://doi.org/10.1159/jvr/adiag003).

### Design and Fabrication of HMC Arrays

HMC array-based imaging plates were fabricated by embossing low-melting-point agarose (LMA) hydrogels onto the inner glass bottom (170 μm thickness) of standard 6- or 24-well plates. Polydimethylsiloxane (PDMS) stamps containing a negative microchamber pattern were generated using silicon oxide masters fabricated by photolithography followed by deep reactive ion etching (Fig. 1).

**Fig. 1.**
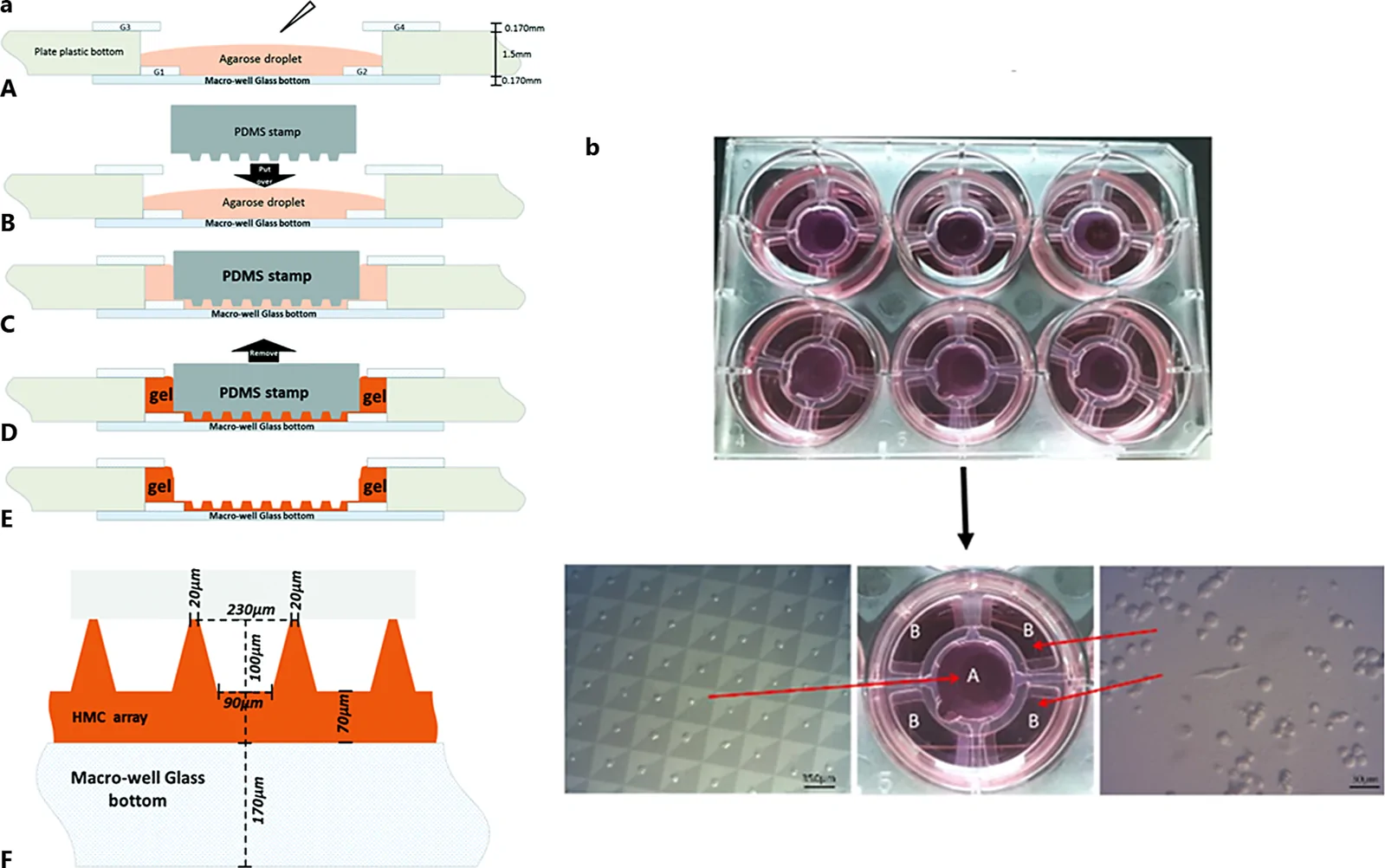
Design and application of the hydrogel microchamber (HMC) array platform. **a** Schematic illustration of HMC fabrication (**A–E**), including deposition of liquid agarose, placement of a PDMS stamp, gelation, and stamp removal, resulting in a patterned hydrogel containing an array of microchambers. A representative design of square microchambers (90 μm) with 250 μm pitch is shown in **F**. **a** reproduced from References [25, 30, 31]. **b** HMC-based imaging plate and representative cell cultures. **A** Modified 6-well imaging plate with each macro-well subdivided into five compartments. **B** Close-up view of a single macro-well. **C** Bright-field image of HUVEC spheroids generated within the HMC platform (scale bar: 350 µm). **D** Bright-field image of fibroblasts within one compartment (region B in **b**) (scale bar: 300 µm).

PDMS precursors were mixed at a 1:9 (w/w) ratio, degassed, poured over the master mold, and cured at 70–80°C for 40 min. LMA was dissolved in PBS to prepare a 5% (w/v) stock solution and maintained at 70°C prior to use. The plates and inserts were plasma-treated twice for 20 s to enhance agarose adhesion. Immediately following plasma treatment, 300 μL of molten LMA was dispensed into each well, and a preheated PDMS stamp was gently placed on top of the solution. After cooling to room temperature and gelation at 4°C for 30 min, the PDMS stamp was removed, leaving a patterned hydrogel surface containing up to 100 truncated square-pyramidal microchambers per well (small base: 90 × 90 μm; large base: 370 × 370 μm; height: ∼190 μm). Plates were subsequently UV sterilized, filled with phosphate-buffered saline (PBS) containing antibiotics, and stored at 4°C until use.

### Cell Culture

Human umbilical vein endothelial cells (HUVECs) were isolated from umbilical cords obtained from healthy women with uncomplicated pregnancies at 38–40 weeks of gestation who delivered at the Obstetrics Unit of Meir Medical Center, Kfar Saba, Israel (Institutional Review Board approval No. 0074-11-MMC). All participants provided written informed consent prior to tissue collection. HUVECs from 3 to 4 independent donors were used across the study, and each experimental repeat was performed using cells derived from a single donor. HUVECs were used at passage 3 and cultured in Medium 199 (M199) supplemented with 20% heat-inactivated fetal bovine serum, heparin, endothelial cell growth supplement, and penicillin-streptomycin. NIH3T3 mouse embryonic fibroblasts were also used at passage 3 and maintained in Dulbecco’s Modified Eagle Medium supplemented with 10% fetal bovine serum, penicillin-streptomycin, L-glutamine, and sodium pyruvate. All cells were maintained at 37°C in a humidified incubator with 5% CO_2_. Prior to seeding into the HMC arrays, cells were detached using trypsin EDTA, washed with PBS, and resuspended in fresh complete medium at the desired cell concentration.

### Formation of Spheroids and Organoids

*For spheroid formation*, cell suspensions (300 μL for 24-well plates or 500 μL for 6-well plates; 1 × 10^5^ cells/mL) were gently loaded onto the HMC arrays and allowed to settle into the microchambers by gravity for 15 min. Fresh culture medium was then gradually added to the periphery of the well to reach a final volume of 2–4 mL.

*For organoid formation*, all procedures were performed at 4°C. After cell settling, the culture medium was carefully removed and replaced with 50% Matrigel diluted in minimally supplemented medium. Plates were subsequently incubated at 37°C for 30 min to allow Matrigel polymerization, followed by the addition of complete culture medium.

### Co-Culture Configurations

Mono-cultures of HUVECs or NIH3T3 fibroblasts, as well as co-culture systems at a 5:1 ratio of HUVECs:NIH3T3, were established. Cells were either loaded into the same central region of the insert or spatially separated, with HUVECs seeded in the central HMC region and fibroblasts in peripheral compartments. This configuration enabled paracrine communication between the cell populations through a shared culture medium.

### Drug Treatment

Microtissues were allowed to mature for 7 days, with partial medium replacement every 3 days. Treatments were applied on day 5 or day 7 according to the experimental design and included four conditions: (1) treatment with each agent individually, (2) simultaneous co-treatment with glucose, insulin, and GLP-1RA (Xultophy), (3) sequential treatment with glucose followed by Xultophy after 24 h, and (4) sequential treatment with Xultophy followed by glucose after 24 h. In sequential conditions, the second treatment was added without removal of the first. Final working concentrations were approximately 13.9 mm glucose and 2 nm each of Xultophy.

### Imaging and Data Analysis

Bright-field images were acquired using an inverted Olympus (IX81) microscope equipped with a BC1200 camera and a 4× objective. Images were analyzed using ImageJ software. Statistical analysis was conducted using SPSS and Microsoft Excel.

Statistical analyses were performed using Microsoft Excel and SPSS version 29 (IBM, Armonk, NY, USA). Data are presented as mean ± SD. Comparisons between treatment groups and the untreated control were performed using Student’s *t* test or an appropriate nonparametric test, depending on data distribution. Because the analyses were exploratory, no formal adjustment for multiple comparisons was applied. Accordingly, all reported *p* values are nominal (unadjusted) and should be interpreted as exploratory rather than confirmatory. Differences were considered statistically significant at *p* < 0.05.

## Results

### Formation and Growth of HUVEC Spheroids and Organoids in HMC Arrays

HUVECs cultured within HMCs reproducibly formed 3 distinct growth configurations depending on culture conditions: conventional monolayers, scaffold-free spheroids, and Matrigel-embedded organoids. In monolayer cultures, HUVECs rapidly adhered to the substrate, spread, and formed a confluent endothelial sheet by day 7 (Fig. 2), with further increases in cell density observed by day 14. Under spheroid-forming conditions in HMCs, HUVECs aggregated within individual microchambers within 24 h, forming compact multicellular spheroids. These spheroids remained spatially confined, exhibited minimal radial expansion, and maintained relatively stable morphology throughout the observation period (days 1–14). In contrast, Matrigel-supported cultures generated organoid-like endothelial structures characterized by progressive growth, increased structural heterogeneity, and irregular contours, consistent with active cellular remodeling and matrix interaction over time (Fig. 2).

**Fig. 2.**
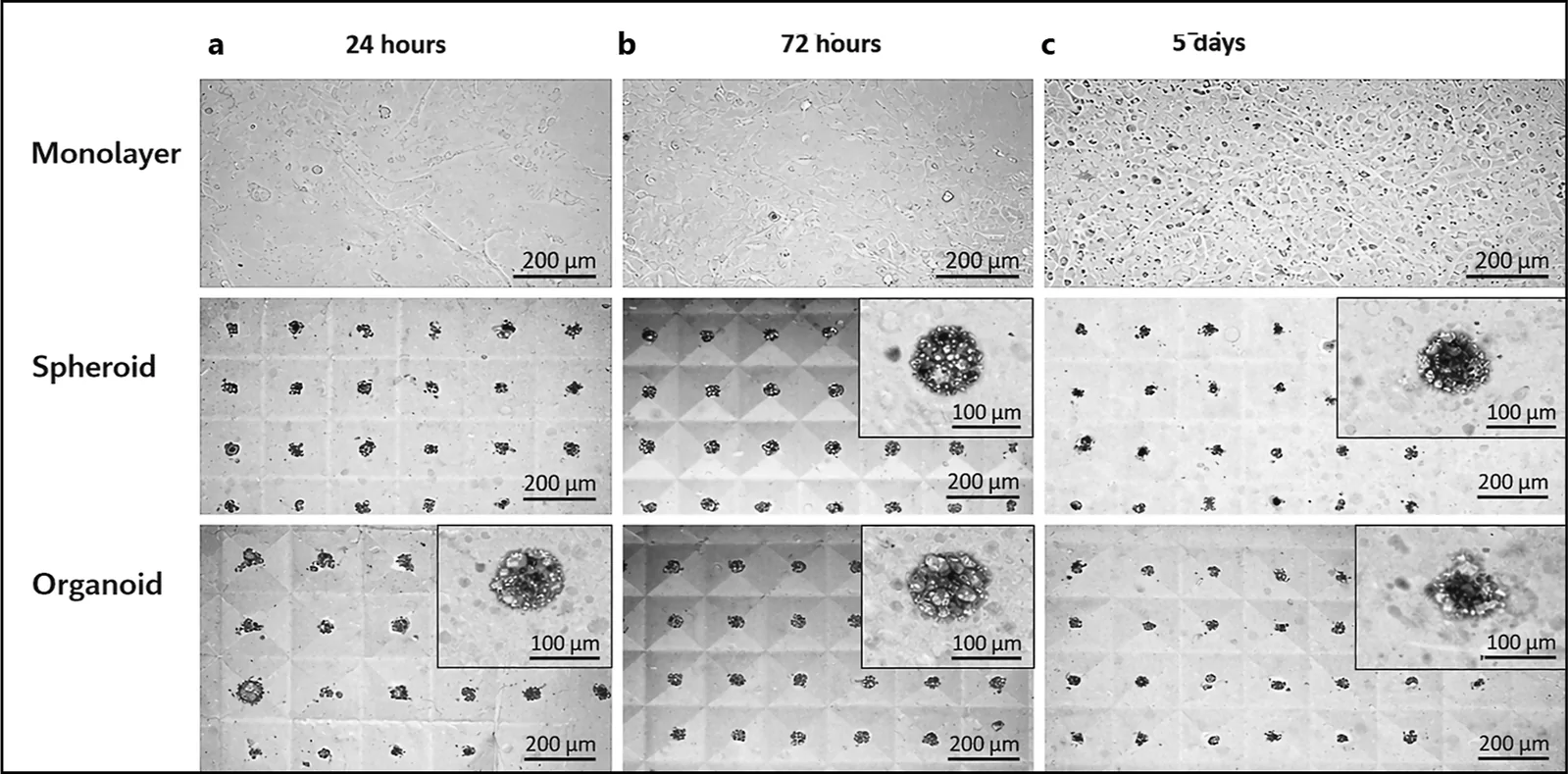
Morphological evolution of HUVEC monolayers, spheroids, and organoids in HMC culture. Representative bright-field images of HUVECs cultured as monolayers (**a**), scaffold-free spheroids (**b**), and Matrigel-embedded organoids (**c**) over time (days 0–14). Monolayers show progressive surface coverage and increased cell density, spheroids remain compact and spatially confined, and organoids exhibit increasing structural heterogeneity. Scale bars: see panels.

To enable direct comparison across these configurations, the HMC platform allowed parallel generation of monolayers, spheroids, and organoids under identical culture conditions. This design facilitated side-by-side evaluation of growth dynamics and morphological evolution across culture formats with increasing structural complexity.

### Impact of Fibroblast Co-Culture on HUVEC 3D Organization

Co-culture of HUVECs with NIH3T3 fibroblasts at a 5:1 ratio markedly altered microtissue morphology compared with HUVEC monocultures. In monolayer co-cultures, fibroblasts promoted rapid formation of interconnected cellular networks accompanied by elongated endothelial morphologies and increased cellular density by days 1–7 (Fig. 3). Within HMCs, co-cultured spheroids exhibited increased compactness and a more uniform size distribution relative to HUVEC-only spheroids. Organoids formed under co-culture conditions demonstrated pronounced radial outgrowth, dense multicellular cores, and extensive peripheral protrusions, indicating enhanced endothelial remodeling and collective migration (Fig. 3). These morphological changes were consistently observed across multiple microchambers within the same well and across independent experiments, suggesting reproducible performance of the HMC platform under the conditions tested.

**Fig. 3.**
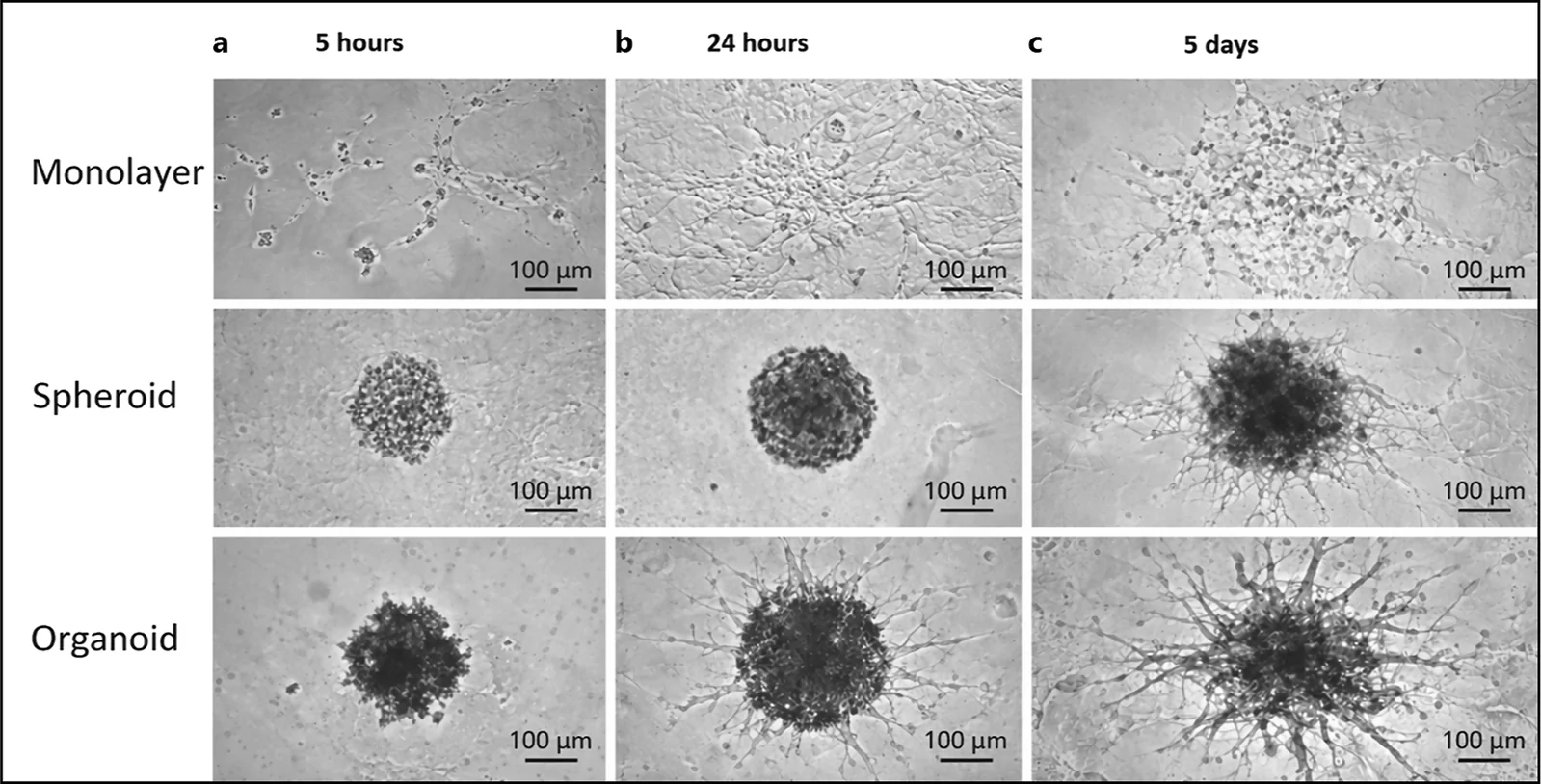
Effect of fibroblast co-culture on HUVEC microtissue organization. Representative bright-field images of HUVECs cultured alone or in co-culture with NIH3T3 fibroblasts (5:1 ratio) as monolayers (**a**), spheroids (**b**), and Matrigel-embedded organoids (**c**). Time points are indicated within each panel. Fibroblast co-culture enhances cellular connectivity in monolayers, increases spheroid compactness, and promotes radial outgrowth and structural complexity in organoids. Scale bars: see panels.

### Quantitative Morphometric Analysis of Organoids following Glucose and Xultophy Treatment

To quantitatively assess the response of co-cultured HUVEC-NIH3T3 organoids to metabolic and hormonal stimulation, morphometric parameters were measured following treatment with glucose, Xultophy, or sequential combinations of both. Organoid area, perimeter, Feret diameter, and circularity were quantified on days 6 and 12 and normalized to pre-treatment values (defined as 100%) to enable comparison across experiments. Unless otherwise indicated, all reported *p* values in this section are unadjusted as no formal correction for multiple comparisons was applied.

Glucose exposure induced marked structural remodeling of organoids, characterized by significant increases in both area and perimeter. Organoid area increased by 217% after 6 days and remained elevated by 137% after 12 days (both *p* < 0.001). Similarly, perimeter increased to approximately 182% and 208% after 6 and 12 days, respectively (both *p* < 0.001). These changes were accompanied by a marked reduction in circularity, which decreased markedly by day 12 (*p* < 0.001), indicating progressive structural expansion and increased morphological complexity. In contrast, Xultophy treatment resulted in only modest morphometric changes that did not reach statistical significance, and organoids maintained a relatively compact and cohesive morphology.

Sequential treatment protocols produced distinct responses. Glucose followed by Xultophy partially attenuated the glucose-induced expansion. In contrast, Xultophy followed by glucose (Xul2-Glu5) resulted in a delayed but pronounced remodeling response, with perimeter increasing by 149% after 6 days and 146% after 12 days (both *p* < 0.001) while circularity decreased to approximately 64% after 12 days (*p* < 0.001). These findings suggest that the sequence of metabolic and pharmacological stimulation influences organoid remodeling dynamics.

Overall, circularity analysis demonstrated that glucose exposure progressively increased morphological irregularity, whereas Xultophy-treated organoids retained higher circularity values. Sequential treatment groups exhibited intermediate phenotypes, indicating that organoid morphology is influenced by both treatment composition and treatment sequence (Fig. 4).

**Fig. 4.**
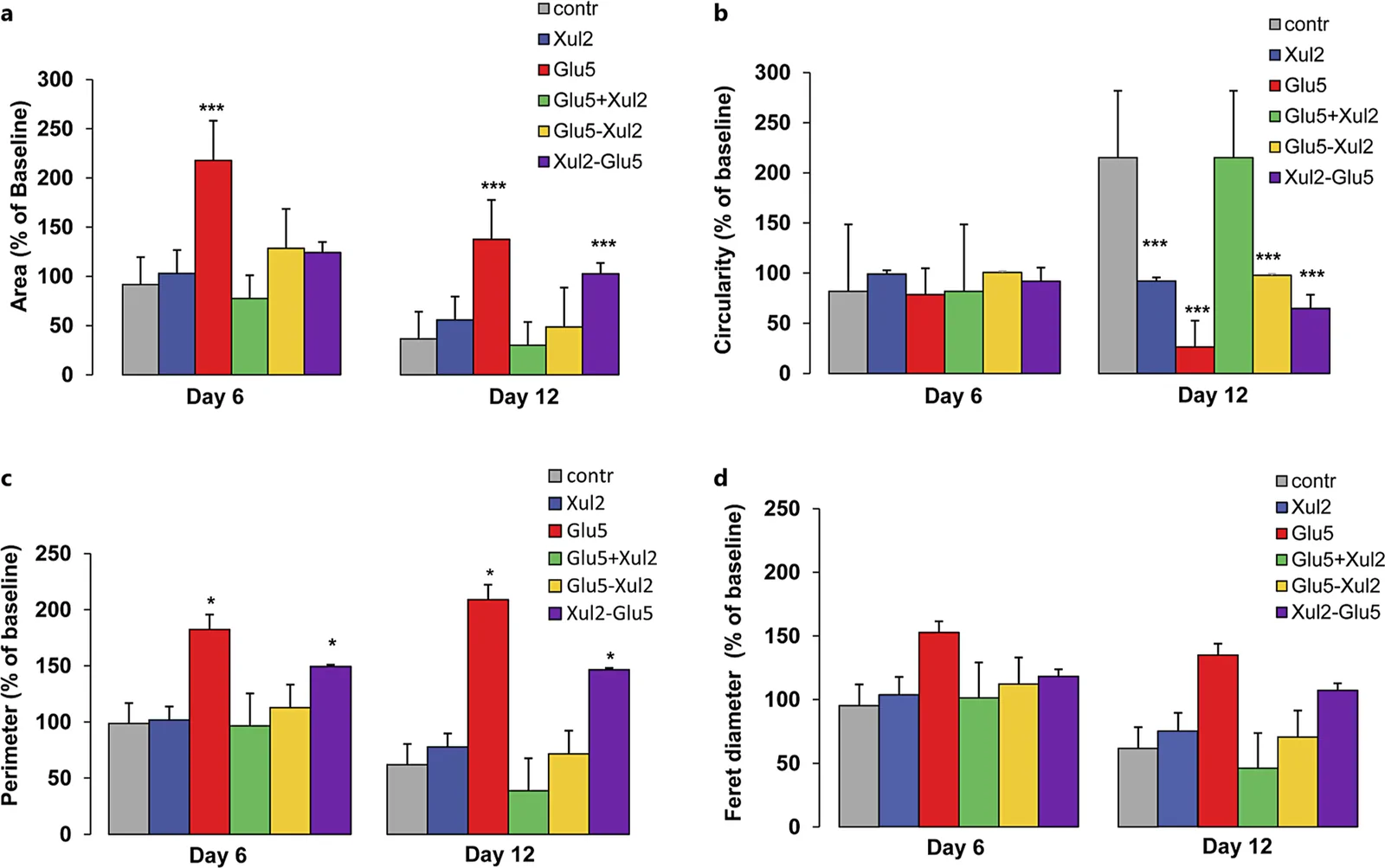
Quantitative morphometric analysis of HUVEC-fibroblast organoids following glucose and Xultophy treatment. **a** Area. **b** Circularity. **c** Perimeter. **d** Feret diameter. Organoid morphology was quantified at days 6 and 12 following exposure to glucose (Glu5), Xultophy (Xul2), or their combinations. Sequential treatments are denoted as Xul2-Glu5 (Xultophy followed by glucose after 24 h) and Glu5-Xul2 (glucose followed by Xultophy after 24 h), whereas Glu5+Xul2 indicates simultaneous co-treatment. “Contr” denotes untreated control. Data are presented as percent change relative to pre-treatment values (defined as 100%). Distinct, sequence-dependent morphometric responses were observed across treatment conditions. Data represent mean ± SD from three independent experiments. *p < 0.05, ***p < 0.001.

### Differential Drug Responses in Spheroids versus Organoids

Comparative analysis revealed distinct differences between spheroids and organoids in their responses to glucose and Xultophy. In co-cultured spheroids, morphometric changes were generally modest compared with those observed in organoids. Glucose treatment induced only limited changes in spheroid area, perimeter, and Feret diameter, while circularity remained largely unchanged throughout the experiment. In contrast to organoid, spheroids exhibited limited structural remodeling and maintained a relatively stable morphology over time (Fig. 5).

**Fig. 5.**
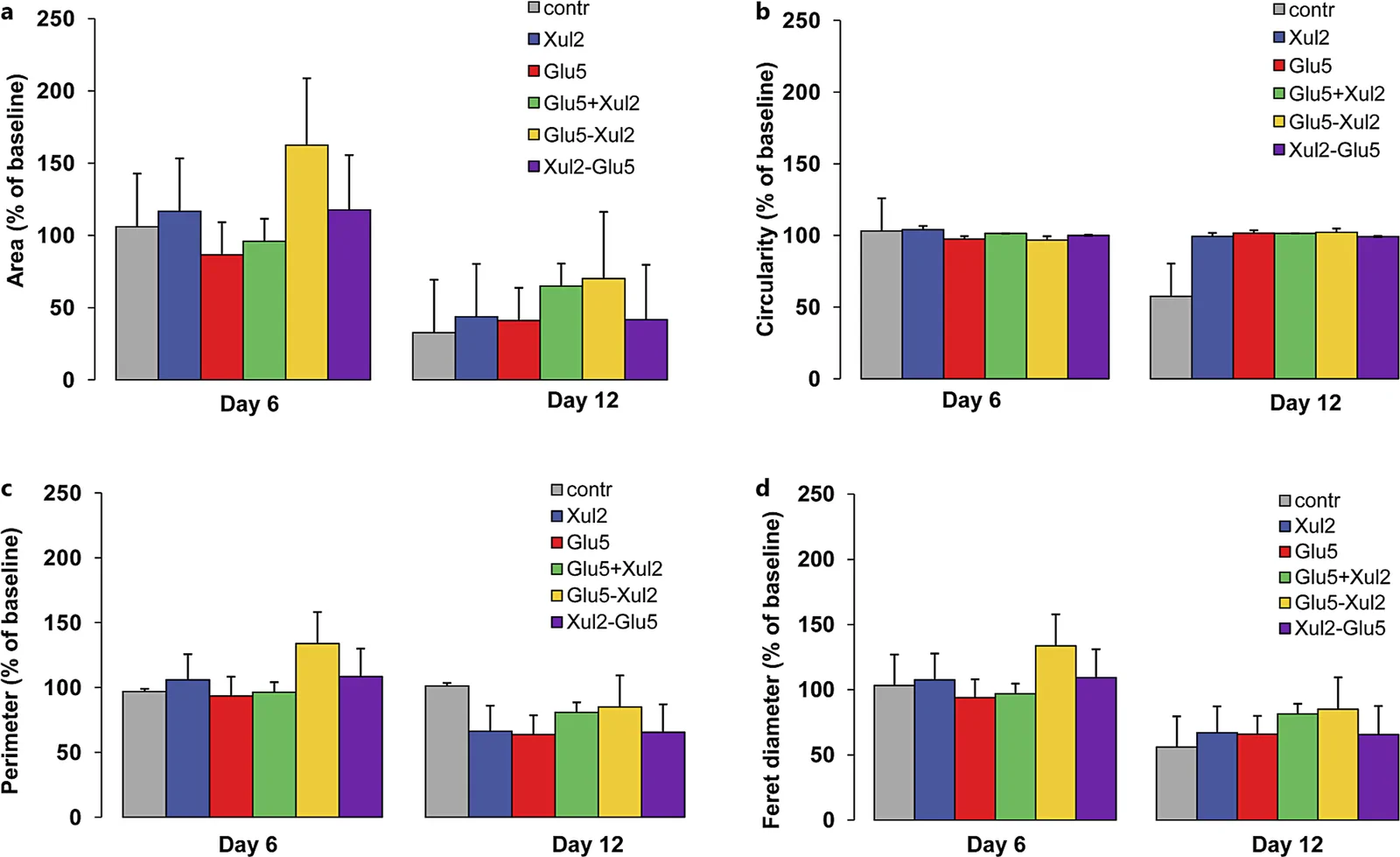
Differential morphometric responses of HUVEC-fibroblast spheroids to glucose and Xultophy treatment. Morphometric parameters of co-cultured spheroids, including area (**a**), circularity (**b**), perimeter (**c**), and Feret diameter (**d**), were analyzed following exposure to glucose (Glu5), Xultophy (Xul2), or their combinations. Sequential treatments are denoted as Xul2-Glu5 (Xultophy followed by glucose after 24 h) and Glu5-Xul2 (glucose followed by Xultophy after 24 h), whereas Glu5+Xul2 indicates simultaneous co-treatment. “Contr” denotes untreated control. Compared to organoids, spheroids exhibit attenuated morphological changes and maintain higher circularity, indicating reduced structural remodeling in the absence of ECM support. Data represent mean ± SD from three independent experiments.

In contrast, Matrigel-embedded organoids exhibited substantially greater remodeling responses than spheroids, including marked alterations in area, perimeter, and circularity following glucose exposure. These findings highlight the critical role of ECM context in regulating endothelial-fibroblast remodeling under metabolic and pharmacological stimulation.

The standardized HMC platform enabled longitudinal analysis of individual microtissues while maintaining consistent initial geometry. Consequently, the observed differences in morphology and treatment responses across culture configurations are likely to reflect biological context rather than technical variability.

## Discussion

This study demonstrated that endothelial structural organization and responsiveness to metabolic and pharmacological stimulation are strongly influenced by culture dimensionality and ECM context. A HMC array platform enabled controlled generation and parallel analysis of endothelial monolayers, spheroids, and organoids derived from HUVECs, within a single experimental framework. By combining spatial confinement with compatibility for both scaffold-free and ECM-supported conditions, the system allows direct comparison of endothelial growth configurations under identical parameters. This approach overcomes a key limitation of conventional in vitro models, where differences in experimental conditions often confound interpretation of structural organization and treatment responses. Consequently, the HMC platform provides a controlled framework for investigating endothelial morphogenesis and environmental regulation of vascular structure (Table 1).

**Table 1.** Comparative characteristics of 3D endothelial culture models

Model	Morphology	Culture conditions	Growth dynamics	Morphological markers	Drug response characteristics
HUVECs spheroids	Compact, smooth spheroids; sprouting upon matrix embedding; partial lumen formation	EGM-2; collagen I, fibrin, Matrigel; VEGF, bFGF	Formation: 24–48 h; sprouting: 2–5 days; stable ∼7 days	Roundness >0.9; sprouting up to 200 µm; 3–10 branches	High sensitivity; rapid response; limited microenvironment complexity
Fibroblast spheroids	Loose, irregular aggregates; ECM-producing; no vascular structures	DMEM + 10% FBS; low-adhesion systems	Formation: 2–4 days; stabilization: 4–7 days	Roundness 0.6–0.8; low compactness; no sprouting	Reflect ECM remodeling; limited endothelial relevance
HUVECs + fibroblast co-spheroids	Multicellular spheroids; endothelial-stromal interaction; network formation	Ratio ∼5:1; mixed medium; collagen I/fibrin; VEGF, FGF	Formation: 24–48 h; sprouting: day 2–3; stable 7–10 days	Roundness >0.85; sprouting 200–400 µm; 5–20 branches	Physiologically relevant; integrates cell-cell and ECM signaling
HUVECs organoid-like	Irregular 3D aggregates; partial lumen formation	Matrigel; VEGF-driven	Formation over several days; limited stability	Variable size; partial lumen; weak organization	Moderate response; improved vs. spheroids
Fibroblast organoid-like	Dense ECM-rich clusters; no lumen; strong remodeling	Collagen-rich matrices	Slow formation; stable	Variable size; high compactness; no branching	Reflect ECM mechanics
HUVECs + fibroblast organoids	Complex 3D structures; vascular-like networks; spatial organization	Mixed-cell; Matrigel/collagen; VEGF, FGF	Formation: several days; maturation 1–2 weeks	Lumen formation; branching; hierarchical structures	High physiological relevance; lower reproducibility

Comparative characteristics of models based on HUVECs, fibroblasts, and their co-culture, including spheroids and organoid-like systems. The table summarizes morphology, culture conditions, growth dynamics, morphometric features, and relevance for drug response modeling.

DMEM, Dulbecco’s Modified Eagle Medium; FBS, fetal bovine serum.

The study was designed to determine whether controlled microchamber-based conditions support consistent formation of endothelial spheroids and organoid-like structures while enabling systematic investigation of stromal regulation and endothelial responses to metabolic and pharmacological stimulation under diabetic-like conditions. HUVECs and fibroblast co-cultures are well-established models of 3D endothelial morphogenesis [17–21] and were used here as a standardized system to validate platform performance. The results demonstrate that the HMC platform supports stable formation of multiple endothelial growth configurations and enables quantitative comparison of their structural dynamics over time.

Clear differences in structural behavior were observed across culture configurations. Endothelial monolayers exhibited progressive surface coverage and increased cell density but remained confined to 2D expansion. In contrast, scaffold-free spheroids formed compact and relatively stable aggregates, whereas matrix-embedded organoids displayed greater structural complexity and dynamic remodeling. These findings emphasize the importance of 3D architecture in modeling endothelial morphogenesis.

A key observation was the divergence between spheroids and organoids. Spheroids maintained a compact morphology with limited remodeling, whereas organoids exhibited pronounced growth dynamics, irregular contours, and increased responsiveness to external stimuli. These differences reflect fundamental distinctions between scaffold-free and ECM-supported culture systems. Spheroids represent simplified 3D models characterized by a structured outer cell layer and central core, which promote survival and stability but limit complex morphogenetic processes [19]. In contrast, ECM-supported organoids enable interactions with matrix components that regulate mechanotransduction, collective migration, tissue organization, and structural plasticity [26–29]. Consistent with previous studies demonstrating matrix-dependent endothelial branching and tissue-like organization [20, 21], the enhanced structural heterogeneity and remodeling observed in organoids within the HMC platform underscore the critical role of matrix-derived cues in enabling higher-order endothelial morphogenesis.

Fibroblast co-culture emerged as an important regulator of endothelial organization. Fibroblasts contribute to vascular morphogenesis through secretion of angiogenic factors such as vascular endothelial growth factor and platelet-derived growth factor, as well as through ECM deposition and remodeling [27–29]. These interactions support endothelial survival, alignment, and stabilization and enhance vessel-like structure formation [19, 29]. In the present system, fibroblast-containing microtissues exhibited enhanced cellular connectivity in monolayers, increased spheroid compactness, and pronounced radial outgrowth in organoids, indicating that stromal signaling and matrix remodeling substantially contribute to endothelial structural plasticity in 3D environments.

Quantitative morphometric analysis further demonstrated that endothelial organoids responded dynamically to metabolic and hormonal stimulation. Glucose exposure induced sustained increases in size-related parameters accompanied by decreased circularity, indicating enhanced growth and structural complexity. In contrast, treatment with GLP-1RA/insulin (Xultophy) resulted in more restrained changes, maintaining a more cohesive structure. Sequential treatment experiments revealed that the order of exposure significantly influenced remodeling trajectories, suggesting that prior metabolic conditioning alters endothelial responsiveness. Such sequence-dependent effects are consistent with evidence that metabolic stress modulates cytoskeletal organization, cell-cell interactions, and matrix engagement, thereby influencing downstream responses to hormonal signaling [28, 29, 32–36]. These findings highlight a temporal dimension of endothelial adaptation, where metabolic history shapes subsequent responses to pharmacological stimulation.

Comparison across 3D culture formats further emphasized the importance of ECM context in mediating treatment responses. While spheroids exhibited measurable morphometric changes, their magnitude was consistently lower than in organoids, likely reflecting limited capacity for structural reorganization in the absence of matrix support. In contrast, organoids integrate mechanical cues, diffusion gradients, and stromal interactions, providing a more physiologically relevant environment for modeling endothelial responses to metabolic and pharmacological perturbations [17, 18, 29].

These observations are particularly relevant within the CKM framework, where endothelial dysfunction and impaired vascular remodeling are central to disease progression [1–7, 27–29, 32–35]. In this study, glucose exposure induced pronounced structural remodeling of endothelial organoids, whereas treatment with GLP-1RA/insulin moderated these changes and preserved a more cohesive architecture. This suggests that metabolic stress and hormonal signaling differentially regulate endothelial structure in 3D environments, consistent with the protective effects of GLP-1RA-based therapies observed clinically. The ability to model these responses in a controlled system provides a valuable tool for investigating mechanisms underlying vascular protection in CKM.

Beyond biological insights, the HMC platform offers several methodological advantages. Conventional spheroid-generation methods often produce heterogeneous aggregates with limited scalability and compatibility with longitudinal imaging [17, 18]. In contrast, microfabricated hydrogel microchambers enable precise control over cell number and microtissue geometry while maintaining optical accessibility and compatibility with standard multi-well formats [30, 31, 36–38]. The system also allows controlled co-culture configurations, including direct contact or shared medium conditions, enabling investigation of both contact-dependent and paracrine stromal signaling. This scalability supports high-content imaging and systematic evaluation of endothelial responses to metabolic and pharmacological perturbations, with potential applications in drug screening.

Collectively, these findings demonstrate that endothelial structural adaptation is governed by the interplay between dimensionality, ECM engagement, and stromal signaling. The ability of the HMC platform to resolve these factors within a controlled system highlights its value for dissecting microenvironment-dependent mechanisms of vascular remodeling.

The platform also has potential translational applications. Its scalability, reproducibility, and compatibility with quantitative imaging make it suitable for high-throughput testing and patient-specific modeling. By enabling controlled assessment of endothelial and stromal responses under defined metabolic and pharmacological conditions, the system may support development of personalized therapeutic strategies and improve understanding of stage-specific mechanisms across the CKM spectrum [1, 2, 5, 30, 31, 36–41].

Several limitations to the study should be considered. It utilized HUVECs and mouse NIH3T3 fibroblasts, and extrapolation to adult human microvascular endothelium requires validation in human and tissue-specific models. In addition, functional endpoints such as barrier integrity, nitric oxide production, oxidative stress, and apoptosis were not assessed. Therefore, conclusions regarding endothelial dysfunction are based on structural remodeling rather than on direct functional measurements. Furthermore, although similar morphological patterns were consistently observed across multiple microchambers and independent experiments using HUVECs derived from 3 to 4 different donors, formal quantitative assessment of reproducibility was not included in the study design. In addition, multiple treatment conditions and morphometric parameters were evaluated without formal adjustment for multiple comparisons. Consequently, the reported *p* values should be considered exploratory and interpreted with appropriate caution. However, the principal findings were consistently associated with highly significant nominal *p* values (*p* < 0.001). Future studies should incorporate a prespecified statistical analysis plan with appropriate multiplicity-adjusted analyses to confirm these observations.

## Conclusions

This study established a HMC array platform for controlled generation and direct comparison of endothelial spheroids and matrix-embedded organoids in mono- and co-culture with fibroblasts. The system revealed that ECM context and treatment sequence are key determinants of endothelial structural remodeling in 3D environments. By enabling standardized, high-resolution analysis of endothelial responses under defined conditions, the HMC platform provides a powerful framework for investigating vascular adaptation to metabolic stress and therapeutic modulation. These findings support the use of physiologically relevant 3D systems for advancing mechanistic understanding and development of targeted therapies in CKM continuum.

## Acknowledgments

This manuscript was written in memory of Mordechai Deutsch, the researcher who established the Endothelial-Fibroblast Organoids laboratory and developed the methodology on which this work is based on.

## Statement of Ethics

Endothelial cells were isolated from umbilical cords obtained from healthy women who completed a normal, uncomplicated pregnancy at 38–40 weeks of gestation, at Meir Medical Center, Kfar Saba, Israel (Institutional Review Board Approval No. 0074-11-MMC). The parturients provided written informed consent. The NIH3T3 fibroblasts used in this study were obtained from Don Laboratory, Department of Cell and Developmental Biology, Mina and Everard Goodman, Faculty of Life Sciences, Bar Ilan University. The use of established cell lines from NIH3T3 fibroblasts in this study did not require approval by the Institutional Ethics Committee, in accordance with institutional and national regulations, as no human subjects or identifiable human-derived materials were involved.

## Conflict of Interest Statement

All authors except for A.D. are employees of Meir Medical Center. K.C.-H. was an invited speaker for AstraZeneca, CTS, Roche, Boehringer Ingelheim, and GSK.

## Funding Sources

This work was funded through the Israeli Ministry of Innovation, Science and Technology (MOST), Science Forefront Grant No. 0006031 and Israel Science Foundation Grant No. 1503/22. The funders had no role in the study design, execution, analysis, manuscript conception, planning, writing, or decision to publish.

## Author Contributions

Research area and study design: T.Z.-G., M.S., M.D., and K.C.-H.; data acquisition: M.S., S.T.-M., G.T., J.C., G.R., A.D., and K.C.-H.; data analysis and interpretation: T.Z.-G., M.S., A.T., D.P., G.R., N.N., A.D., M.D., and K.C.-H.; statistical analysis: T.Z.-G., M.S., A.T., and K.C.-H.; supervision or mentorship: T.Z.-G., A.D., M.D., and K.C.-H. Each author contributed important intellectual content during manuscript drafting or revision and agrees to be personally accountable for the individual’s own contributions.

## Data Availability

All data generated or analyzed during this study are included in this article and its online supplementary material. Further inquiries can be directed to the corresponding author.
